# Supplementary material for: Structure of the hibernating Francisella tularensis ribosome and mechanistic insights into its inhibition by antibiotics
Source: Nucleic Acids Res. 2026 Apr 28;54(8):gkag340. doi: 10.1093/nar/gkag340 (PMC13122176; doi:10.1093/nar/gkag340)
Supplement: gkag340_Supplemental_File [file gkag340_supplemental_file.pdf]

Structure of the Hibernating *Francisella tularensis* Ribosome and Mechanistic Insights into Its Inhibition by Antibiotics

Martin Klima[a,#], Jan Silhan[a,#], Pavla Pavlik[a,b], Kamil Hercik[a], Evzen Boura[a,*]

[a]Institute of Organic Chemistry and Biochemistry, Academy of Sciences of the Czech Republic, v.v.i, Flemingovo namesti 2, Prague 6, 16600, Czech Republic.

[b]Military Faculty of Medicine, University of Defence, Trebesska 1575, Hradec Kralove 50001, Czech Republic

[#] these authors contributed equally

[*] correspondence to and boura@uochb.cas.cz

a

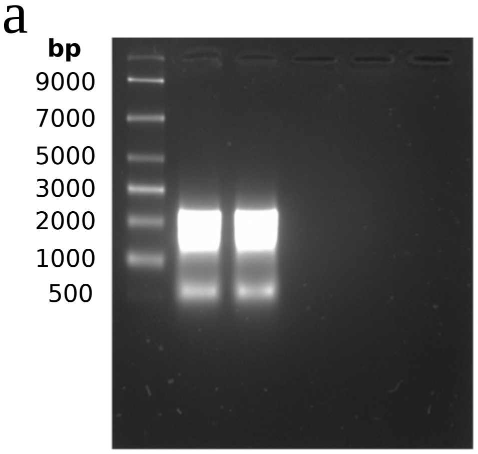

b

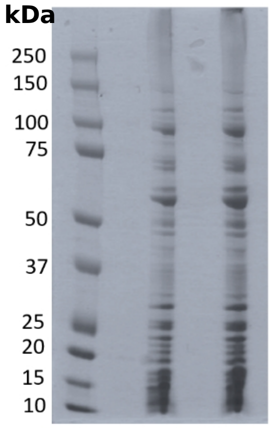

c

**Alignment results link**		[**CLICK HERE**]						
**Entry**	**Reviewed**	**Entry Name**	**Protein names**	**Gene Names**	**Organism**	**Length**	**Identity (Pairwise Sequence Alignment) SCHU S4 vs FSC200 (%)**	**Similarity (Pairwise Sequence Alignment) SCHU S4 vs FSC200 (%)**
Q5NID3	reviewed	RL7_FRATT	Large ribosomal subunit protein bL12 (50S ribosomal protein L7/L12)	rplL FTT_0143	Francisella tularensis subsp. tularensis (strain SCHU S4 / Schu 4)	125	98,4	98,4
Q5NHU2	reviewed	RL17_FRATT	Large ribosomal subunit protein bL17 (50S ribosomal protein L17)	rplQ FTT_0351	Francisella tularensis subsp. tularensis (strain SCHU S4 / Schu 4)	145	99,3	99,3
Q5NIC3	reviewed	RL19_FRATT	Large ribosomal subunit protein bL19 (50S ribosomal protein L19)	rplS FTT_0153	Francisella tularensis subsp. tularensis (strain SCHU S4 / Schu 4)	115	98,3	99,1
Q5NGL5	reviewed	RL20_FRATT	Large ribosomal subunit protein bL20 (50S ribosomal protein L20)	rplT FTT_0820	Francisella tularensis subsp. tularensis (strain SCHU S4 / Schu 4)	118	98,3	98,3
Q5NGR2	reviewed	RL21_FRATT	Large ribosomal subunit protein bL21 (50S ribosomal protein L21)	rplU FTT_0772	Francisella tularensis subsp. tularensis (strain SCHU S4 / Schu 4)	104	99	99
Q5NH01	reviewed	RL25_FRATT	Large ribosomal subunit protein bL25 (50S ribosomal protein L25)	rplY FTT_0675	Francisella tularensis subsp. tularensis (strain SCHU	96	99	99

					S4 / Schu 4)			
Q5NGR1	reviewed	RL27_FRATT	Large ribosomal subunit protein bL27 (50S ribosomal protein L27)	rpmA FTT_0773	Francisella tularensis subsp. tularensis (strain SCHU S4 / Schu 4)	84	98,8	98,8
Q5NEM3	reviewed	RL28_FRATT	Large ribosomal subunit protein bL28 (50S ribosomal protein L28)	rpmB FTT_1603	Francisella tularensis subsp. tularensis (strain SCHU S4 / Schu 4)	78	100	100
Q5NHS9	reviewed	RL31_FRATT	Large ribosomal subunit protein bL31 (50S ribosomal protein L31)	rpmE FTT_0366	Francisella tularensis subsp. tularensis (strain SCHU S4 / Schu 4)	71	97,2	97,2
Q5NF72	reviewed	RL32_FRATT	Large ribosomal subunit protein bL32 (50S ribosomal protein L32)	rpmF FTT_1371	Francisella tularensis subsp. tularensis (strain SCHU S4 / Schu 4)	60	98,3	100
Q5NEM2	reviewed	RL33_FRATT	Large ribosomal subunit protein bL33 (50S ribosomal protein L33)	rpmG FTT_1604	Francisella tularensis subsp. tularensis (strain SCHU S4 / Schu 4)	51	100	100
Q5NI53	reviewed	RL34_FRATT	Large ribosomal subunit protein bL34 (50S ribosomal protein L34)	rpmH FTT_0236c	Francisella tularensis subsp. tularensis (strain SCHU S4 / Schu 4)	44	100	100
Q5NGL6	reviewed	RL35_FRATT	Large ribosomal subunit protein bL35 (50S ribosomal protein L35)	rpmI FTT_0819	Francisella tularensis subsp. tularensis (strain SCHU S4 / Schu 4)	65	100	100
Q5NHU7	reviewed	RL36_FRATT	Large ribosomal subunit protein bL36 (50S ribosomal protein L36)	rpmJ FTT_0346	Francisella tularensis subsp. tularensis (strain SCHU S4 / Schu 4)	37	100	100
Q5NG01	reviewed	RL9_FRATT	Large ribosomal subunit protein bL9 (50S ribosomal protein L9)	rplI FTT_1060c	Francisella tularensis subsp. tularensis (strain SCHU S4 / Schu 4)	151	99,3	99,3
Q5NID5	reviewed	RL1_FRATT	Large ribosomal subunit protein uL1 (50S ribosomal protein L1)	rplA FTT_0141	Francisella tularensis subsp. tularensis (strain SCHU S4 / Schu 4)	231	99,6	100
Q5NID4	reviewed	RL10_FRATT	Large ribosomal subunit protein uL10 (50S ribosomal protein L10)	rplJ FTT_0142	Francisella tularensis subsp. tularensis (strain SCHU S4 / Schu 4)	172	98,8	100
Q5NID6	reviewed	RL11_FRATT	Large ribosomal subunit protein uL11 (50S ribosomal	rplK FTT_0140	Francisella tularensis subsp. tularensis	144	99,3	100

			protein L11)		(strain SCHU S4 / Schu 4)			
Q5NFG3	unreviewed	Q5NFG3_FRATT	Large ribosomal subunit protein uL13	rplM FTT_1273	Francisella tularensis subsp. tularensis (strain SCHU S4 / Schu 4)	151	94	94
Q5NHV8	reviewed	RL14_FRATT	Large ribosomal subunit protein uL14 (50S ribosomal protein L14)	rplN FTT_0335	Francisella tularensis subsp. tularensis (strain SCHU S4 / Schu 4)	122	100	100
Q5NHU9	reviewed	RL15_FRATT	Large ribosomal subunit protein uL15 (50S ribosomal protein L15)	rplO FTT_0344	Francisella tularensis subsp. tularensis (strain SCHU S4 / Schu 4)	143	98,6	98,6
Q5NHW1	reviewed	RL16_FRATT	Large ribosomal subunit protein uL16 (50S ribosomal protein L16)	rplP FTT_0332	Francisella tularensis subsp. tularensis (strain SCHU S4 / Schu 4)	137	99,3	100
Q5NHV2	reviewed	RL18_FRATT	Large ribosomal subunit protein uL18 (50S ribosomal protein L18)	rplR FTT_0341	Francisella tularensis subsp. tularensis (strain SCHU S4 / Schu 4)	117	100	100
Q5NHW5	reviewed	RL2_FRATT	Large ribosomal subunit protein uL2 (50S ribosomal protein L2)	rplB FTT_0328	Francisella tularensis subsp. tularensis (strain SCHU S4 / Schu 4)	274	99,6	100
Q5NHW3	reviewed	RL22_FRATT	Large ribosomal subunit protein uL22 (50S ribosomal protein L22)	rplV FTT_0330	Francisella tularensis subsp. tularensis (strain SCHU S4 / Schu 4)	111	100	100
Q5NHW6	reviewed	RL23_FRATT	Large ribosomal subunit protein uL23 (50S ribosomal protein L23)	rplW FTT_0327	Francisella tularensis subsp. tularensis (strain SCHU S4 / Schu 4)	99	99	99
Q5NHV7	reviewed	RL24_FRATT	Large ribosomal subunit protein uL24 (50S ribosomal protein L24)	rplX FTT_0336	Francisella tularensis subsp. tularensis (strain SCHU S4 / Schu 4)	105	99	99
Q5NHW0	reviewed	RL29_FRATT	Large ribosomal subunit protein uL29 (50S ribosomal protein L29)	rpmC FTT_0333	Francisella tularensis subsp. tularensis (strain SCHU S4 / Schu 4)	66	98,5	98,5
Q5NHW8	reviewed	RL3_FRATT	Large ribosomal subunit protein uL3 (50S ribosomal protein L3)	rplC FTT_0325	Francisella tularensis subsp. tularensis (strain SCHU S4 / Schu 4)	211	99,5	99,5
Q5NHV0	reviewed	RL30_FRATT	Large ribosomal subunit protein uL30 (50S	rpmD FTT_0343	Francisella tularensis subsp.	61	100	100

			ribosomal protein L30)		tularensis (strain SCHU S4 / Schu 4)			
Q5NHW7	reviewed	RL4_FRATT	Large ribosomal subunit protein uL4 (50S ribosomal protein L4)	rplD FTT_0326	Francisella tularensis subsp. tularensis (strain SCHU S4 / Schu 4)	207	100	100
Q5NHV6	reviewed	RL5_FRATT	Large ribosomal subunit protein uL5 (50S ribosomal protein L5)	rplE FTT_0337	Francisella tularensis subsp. tularensis (strain SCHU S4 / Schu 4)	179	99,4	99,4
Q5NHV3	reviewed	RL6_FRATT	Large ribosomal subunit protein uL6 (50S ribosomal protein L6)	rplF FTT_0340	Francisella tularensis subsp. tularensis (strain SCHU S4 / Schu 4)	178	99,4	100
Q5NI98	unreviewed	Q5NI98_FRATT	Small ribosomal subunit protein bS1 (30S ribosomal protein S1)	rpsA FTT_0183c	Francisella tularensis subsp. tularensis (strain SCHU S4 / Schu 4)	556	99,5	99,6
Q5NIC6	reviewed	RS16_FRATT	Small ribosomal subunit protein bS16 (30S ribosomal protein S16)	rpsP FTT_0150	Francisella tularensis subsp. tularensis (strain SCHU S4 / Schu 4)	82	98,8	98,8
Q5NG00	reviewed	RS18_FRATT	Small ribosomal subunit protein bS18 (30S ribosomal protein S18)	rpsR FTT_1061c	Francisella tularensis subsp. tularensis (strain SCHU S4 / Schu 4)	72	97,2	98,6
Q5NEF7	reviewed	RS20_FRATT	Small ribosomal subunit protein bS20 (30S ribosomal protein S20)	rpsT FTT_1679	Francisella tularensis subsp. tularensis (strain SCHU S4 / Schu 4)	90	98,9	98,9
Q5NHQ7	reviewed	RS211_FRATT	Small ribosomal subunit protein bS21A (30S ribosomal protein S21 1)	rpsU1 FTT_0390c	Francisella tularensis subsp. tularensis (strain SCHU S4 / Schu 4)	65	96,9	98,5
Q5NGS8	reviewed	RS212_FRATT	Small ribosomal subunit protein bS21B (30S ribosomal protein S21 2)	rpsU2 FTT_0753	Francisella tularensis subsp. tularensis (strain SCHU S4 / Schu 4)	66	100	100
Q5NG21	reviewed	RS213_FRATT	Small ribosomal subunit protein bS21C (30S ribosomal protein S21 3)	rpsU3 FTT_1038c	Francisella tularensis subsp. tularensis (strain SCHU S4 / Schu 4)	65	100	100
Q5NFZ9	reviewed	RS6_FRATT	Small ribosomal subunit protein bS6 (30S ribosomal protein S6)	rpsF FTT_1062c	Francisella tularensis subsp. tularensis (strain SCHU S4 / Schu 4)	111	100	100
Q5NHW9	reviewed	RS10_FRATT	Small ribosomal subunit protein	rpsJ FTT_032	Francisella tularensis	105	99	100

			uS10 (30S ribosomal protein S10)	4	subsp. tularensis (strain SCHU S4 / Schu 4)			
Q5NHU5	reviewed	RS11_FRATT	Small ribosomal subunit protein uS11 (30S ribosomal protein S11)	rpsK FTT_0348	Francisella tularensis subsp. tularensis (strain SCHU S4 / Schu 4)	129	98,4	99,2
Q5NHX2	reviewed	RS12_FRATT	Small ribosomal subunit protein uS12 (30S ribosomal protein S12)	rpsL FTT_0321	Francisella tularensis subsp. tularensis (strain SCHU S4 / Schu 4)	124	100	100
Q5NHU6	reviewed	RS13_FRATT	Small ribosomal subunit protein uS13 (30S ribosomal protein S13)	rpsM FTT_0347	Francisella tularensis subsp. tularensis (strain SCHU S4 / Schu 4)	118	100	100
Q5NHV5	reviewed	RS14_FRATT	Small ribosomal subunit protein uS14 (30S ribosomal protein S14)	rpsN FTT_0338	Francisella tularensis subsp. tularensis (strain SCHU S4 / Schu 4)	101	100	100
Q5NGX8	reviewed	RS15_FRATT	Small ribosomal subunit protein uS15 (30S ribosomal protein S15)	rpsO FTT_0698	Francisella tularensis subsp. tularensis (strain SCHU S4 / Schu 4)	88	98,9	100
Q5NHV9	reviewed	RS17_FRATT	Small ribosomal subunit protein uS17 (30S ribosomal protein S17)	rpsQ FTT_0334	Francisella tularensis subsp. tularensis (strain SCHU S4 / Schu 4)	83	98,8	98,8
Q5NHW4	reviewed	RS19_FRATT	Small ribosomal subunit protein uS19 (30S ribosomal protein S19)	rpsS FTT_0329	Francisella tularensis subsp. tularensis (strain SCHU S4 / Schu 4)	92	100	100
Q5NHY0	reviewed	RS2_FRATT	Small ribosomal subunit protein uS2 (30S ribosomal protein S2)	rpsB FTT_0313	Francisella tularensis subsp. tularensis (strain SCHU S4 / Schu 4)	239	98,7	98,7
Q5NHW2	reviewed	RS3_FRATT	Small ribosomal subunit protein uS3 (30S ribosomal protein S3)	rpsC FTT_0331	Francisella tularensis subsp. tularensis (strain SCHU S4 / Schu 4)	222	98,2	98,7
Q5NHU4	reviewed	RS4_FRATT	Small ribosomal subunit protein uS4	rpsD FTT_0349	Francisella tularensis subsp. tularensis (strain SCHU S4 / Schu 4)	206	100	100
Q5NHV1	reviewed	RS5_FRATT	Small ribosomal subunit protein uS5 (30S ribosomal protein S5)	rpsE FTT_0342	Francisella tularensis subsp. tularensis (strain SCHU S4 / Schu 4)	166	99,4	100
Q5NHX	reviewed	RS7_FRATT	Small ribosomal	rpsG	Francisella	157	76,4	76,4

1			subunit protein uS7 (30S ribosomal protein S7)	FTT_0322	tularensis subsp. tularensis (strain SCHU S4 / Schu 4)			
Q5NHV4	reviewed	RS8_FRATT	Small ribosomal subunit protein uS8 (30S ribosomal protein S8)	rpsH FTT_0339	Francisella tularensis subsp. tularensis (strain SCHU S4 / Schu 4)	132	99,2	100
Q5NFG2	unreviewed	Q5NFG2_FRATT	Small ribosomal subunit protein uS9 (30S ribosomal protein S9)	rpsI FTT_1274	Francisella tularensis subsp. tularensis (strain SCHU S4 / Schu 4)	132	100	100
				**Average percentage of identity/similarity**			**98,71785714**	**98,975**
**rRNA alignment**								
**Locus tag**	**Locus tag**	**Locus tag**	**Locus tag**	**Gene**	**Strain**		**Identity (Pairwise Sequence Alignment) SCHU S4 vs FSC200 (%)**	**Similarity (Pairwise Sequence Alignment) SCHU S4 vs FSC200 (%)**
FTT_r10	FTT_r04	FTT_r07		16S rRNA	Francisella tularensis subsp. tularensis (strain SCHU S4 / Schu 4)		99,3	99,3
FTS_1142	FTS_0441	FTS_0114		16S rRNA	Francisella tularensis subsp. holarctica (strain FSC200)			
FTT_r09	FTT_r06	FTT_r03		23S rRNA	Francisella tularensis subsp. tularensis (strain SCHU S4 / Schu 4)		98,7	98,7
FTS_1145	FTS_0444	FTS_0117		23S rRNA	Francisella tularensis subsp. holarctica (strain FSC200)			
FTT_r01	FTT_r02	FTT_r05	FTT_r08	5S rRNA	Francisella tularensis subsp. tularensis (strain SCHU S4 / Schu 4)		95,7	95,7
FTS_1146	FTS_0475	FTS_0445	FTS_0118	5S rRNA	Francisella tularensis subsp.			

					holarctica (strain FSC200)			
**Average percentage of identity/similarity**							**97,9**	**97,9**

SI Figure 1. RNA electrophoresis, SDS-PAGE, and Pairwise alignment of ribosomal subunits
a RNA electrophoresis. RNA content in ribosome samples was analyzed by agarose gel electrophoresis. Samples were mixed with RNA loading dye (New England Biolabs) and loaded onto a 1% agarose gel. Electrophoresis was performed at 100 V for 30 min, and RNA bands were visualized under UV illumination. 1. line – ladder, 2. and 3. lines – two replicates of ribosome samples
b Protein electrophoresis. Protein content in ribosome preparations was analyzed by SDS–polyacrylamide gel electrophoresis (SDS–PAGE). Samples were loaded onto a 12% polyacrylamide gel and electrophoresed at 200 V for 45 min. 1. line – ladder, 2. and 3. lines – two replicates of ribosome samples
c Pairwise alignment of ribosomal subunits FSC200 vs. Schu S4. Each subunit of FSC200 and Schu S4 ribosome was aligned using pairwise sequence alignment tool available online at: www.ebi.ac.uk/jdispatcher/psa/emboss_needle

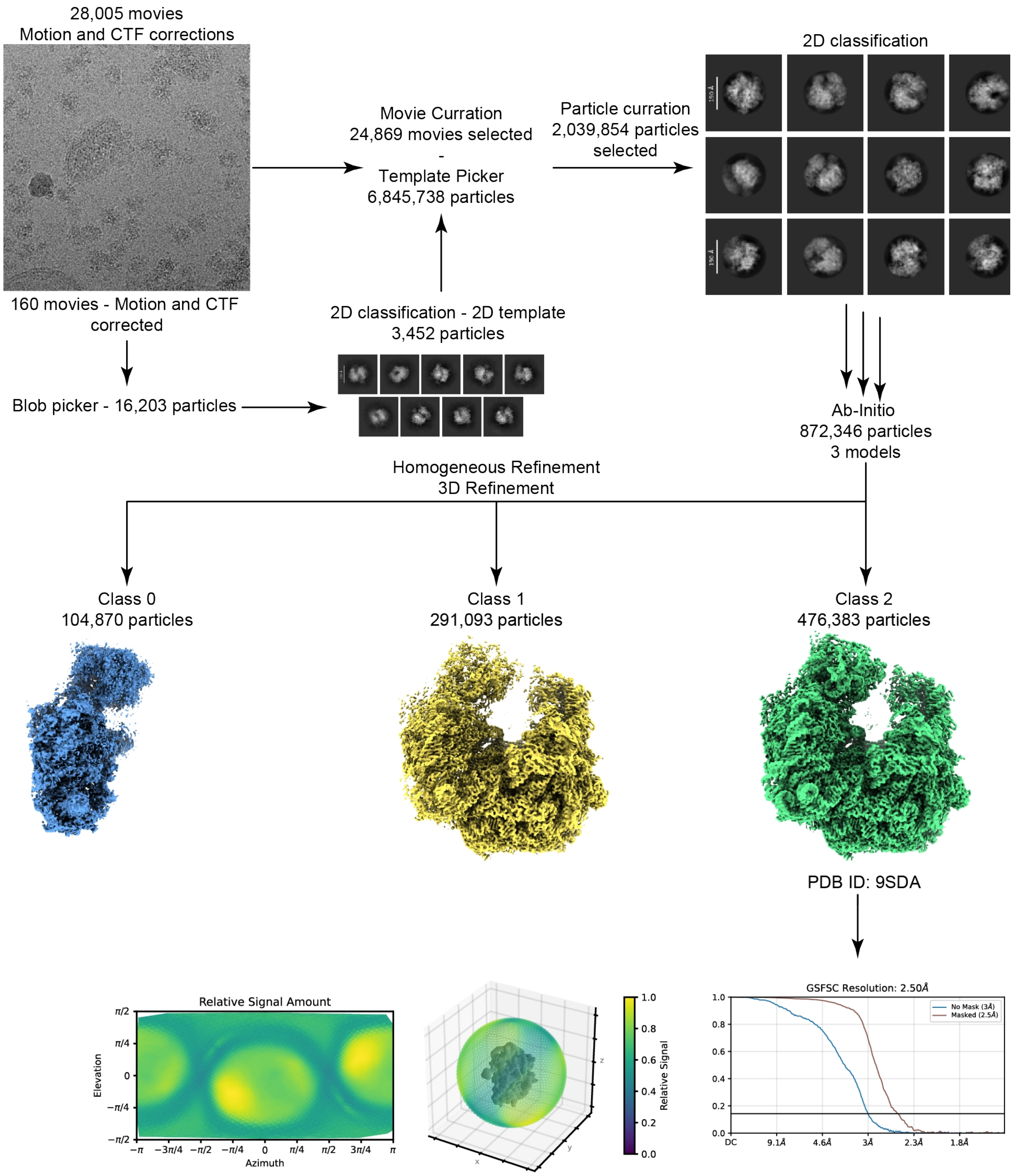

SI Figure 2. Workflow *F. tularensis* 70S

Cryo-EM processing from raw movies collected on Titan Krios with Falcon 4i detector containing sample with *F. tularensis* 70S. Straightforward cryosparc processing lead to final 3D reconstruction with GSFSC final resolution at 2.5 Å.

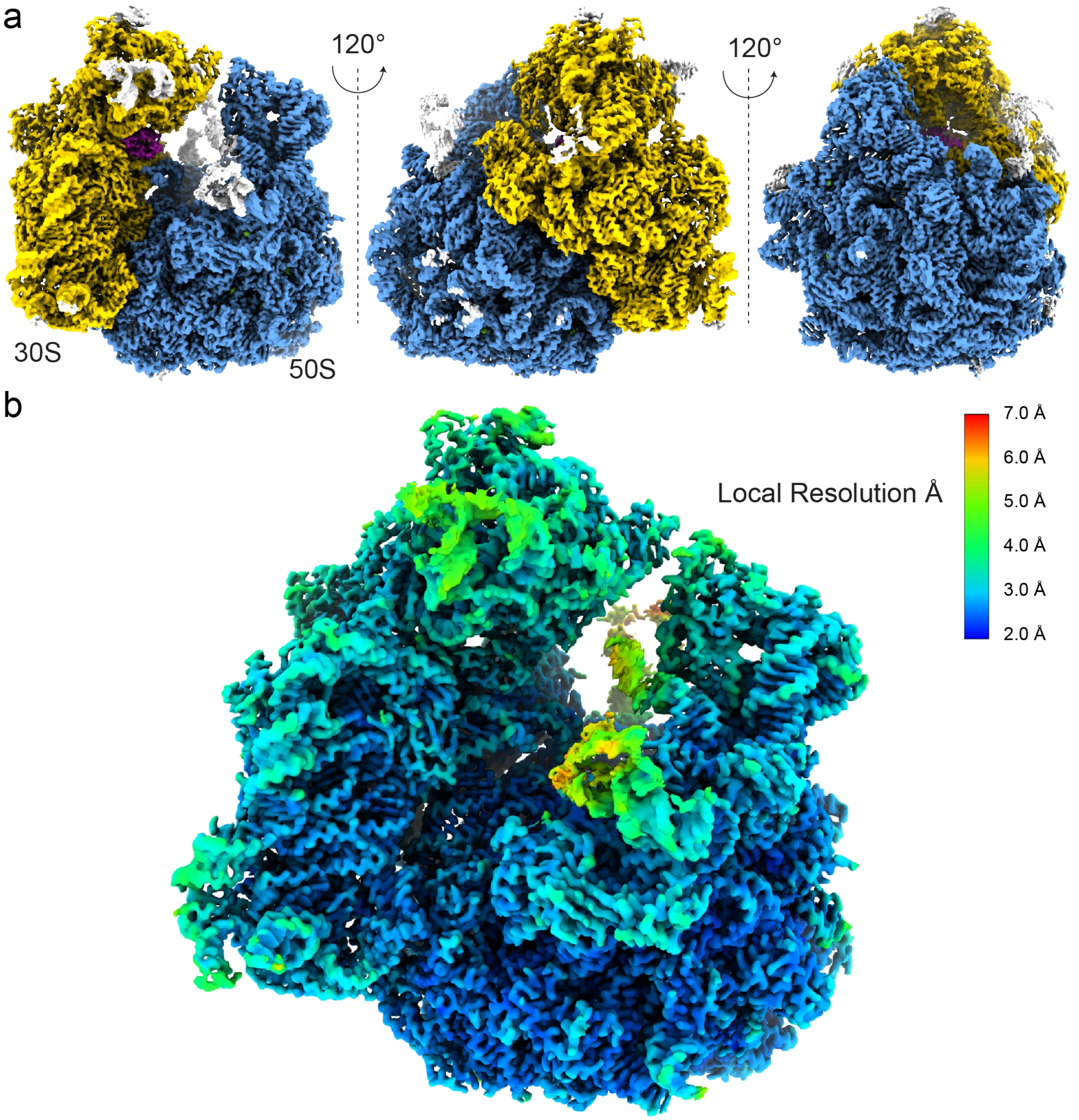

SI Figure 3. CryoEM maps of 70S ribosome
a Cryo-EM maps of *F. tularensis* 70S ribosome coloured by subunits; 30S yellow, 50S blue, and RaiA violet. Maps with poor density that did not allow model building are shown in red.
b Cryo-EM map coloured by local resolution estimation.

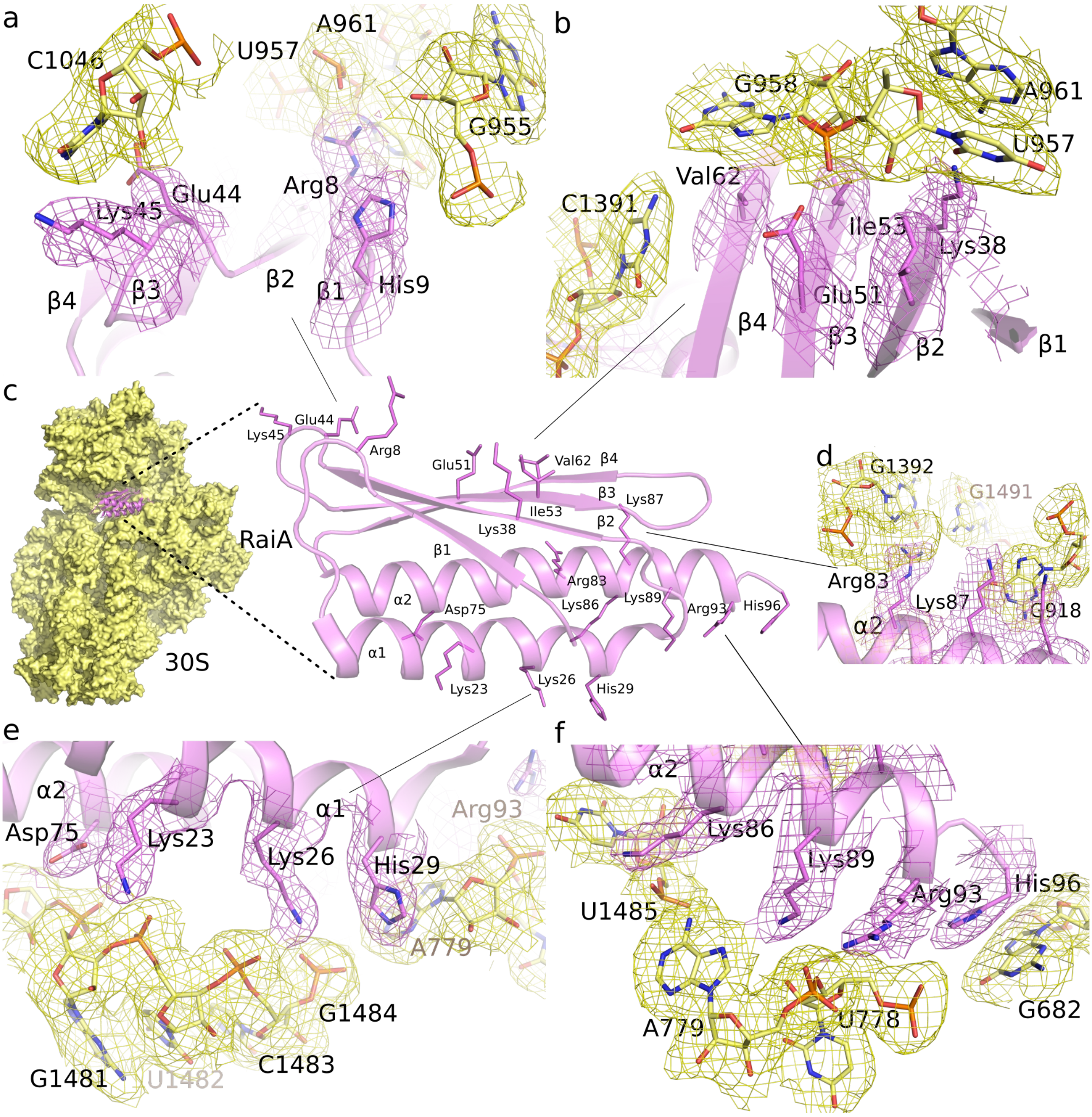

SI Figure 4. Cryo-EM maps detailing the Rai – 30S interaction

a, b, d–f, Close-up views of residues mediating contacts between RaiA (violet) and the 16S rRNA of the 30S subunit (yellow). For clarity, only the residues within an interaction distance of 3.6 Å are shown, along with the cryo-EM map in their vicinity, coloured according to the residues (maps are contoured at σ = 2.0). Carbon atoms are coloured according to their respective chains. Surface of the 30S subunit (yellow) with RaiA (violet) is shown. **e, f,** Two rows of highly conserved charged residues located on helices α1 and α2 interact predominantly with the phosphate backbone of the 16S rRNA.

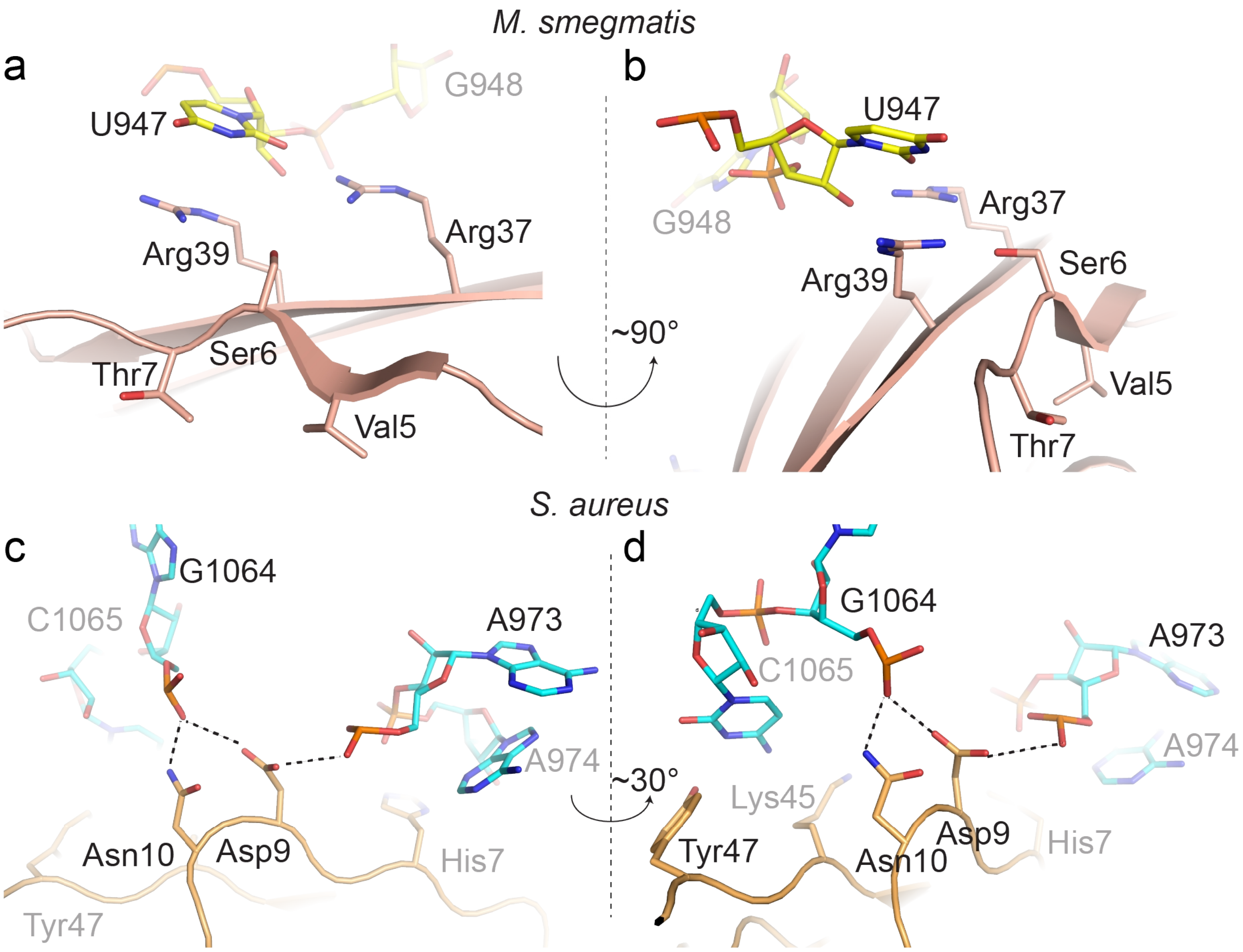

SI Figure 5. Details of the interactions of RaiA homologues within 70S ribosomes from different bacterial species.
a–b, Detail of the interaction between Arg39 of RafH and U947 of the 16S rRNA from *M. smegmatis* (PDB ID: 8WHX).
c–d, Details of the interaction of the hibernation-promoting factor (HPF/YfiA family) from *Staphylococcus aureus*, showing Asp9 and Asn10 in contact with the 16S rRNA (PDB ID: 6S0X).

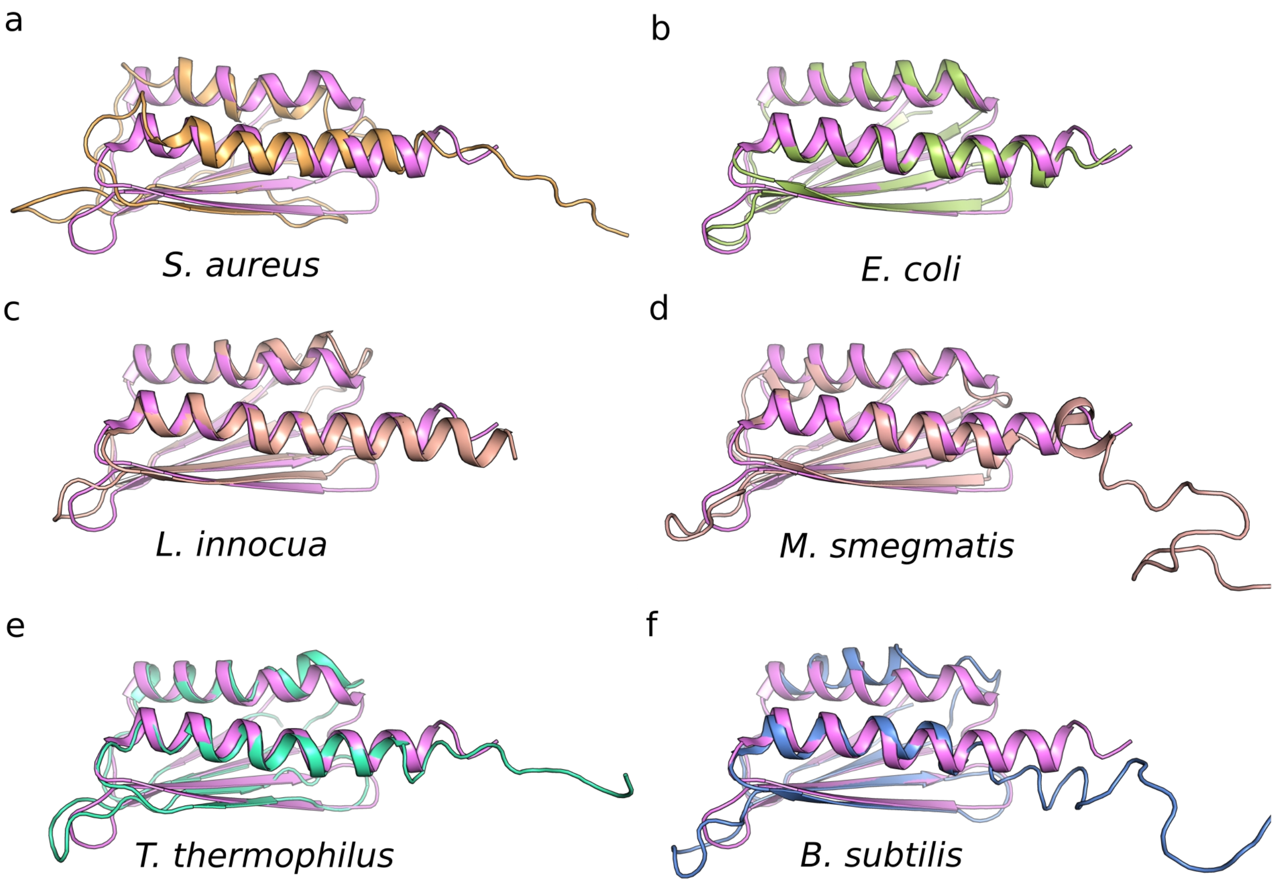

SI Figure 6. Structural alignment of *F. tularensis* RaiA with differences in Ribosome Hiberenation Factors among representative species
alignment *of F. tularensis RaiA* with **a** *S. aureus*, **b** *E. coli*, **c** *Listeria innocua*, **d** *M. smegmatis* RafH, **e** *B. subtilis*, **f** *T. thermophilus.*

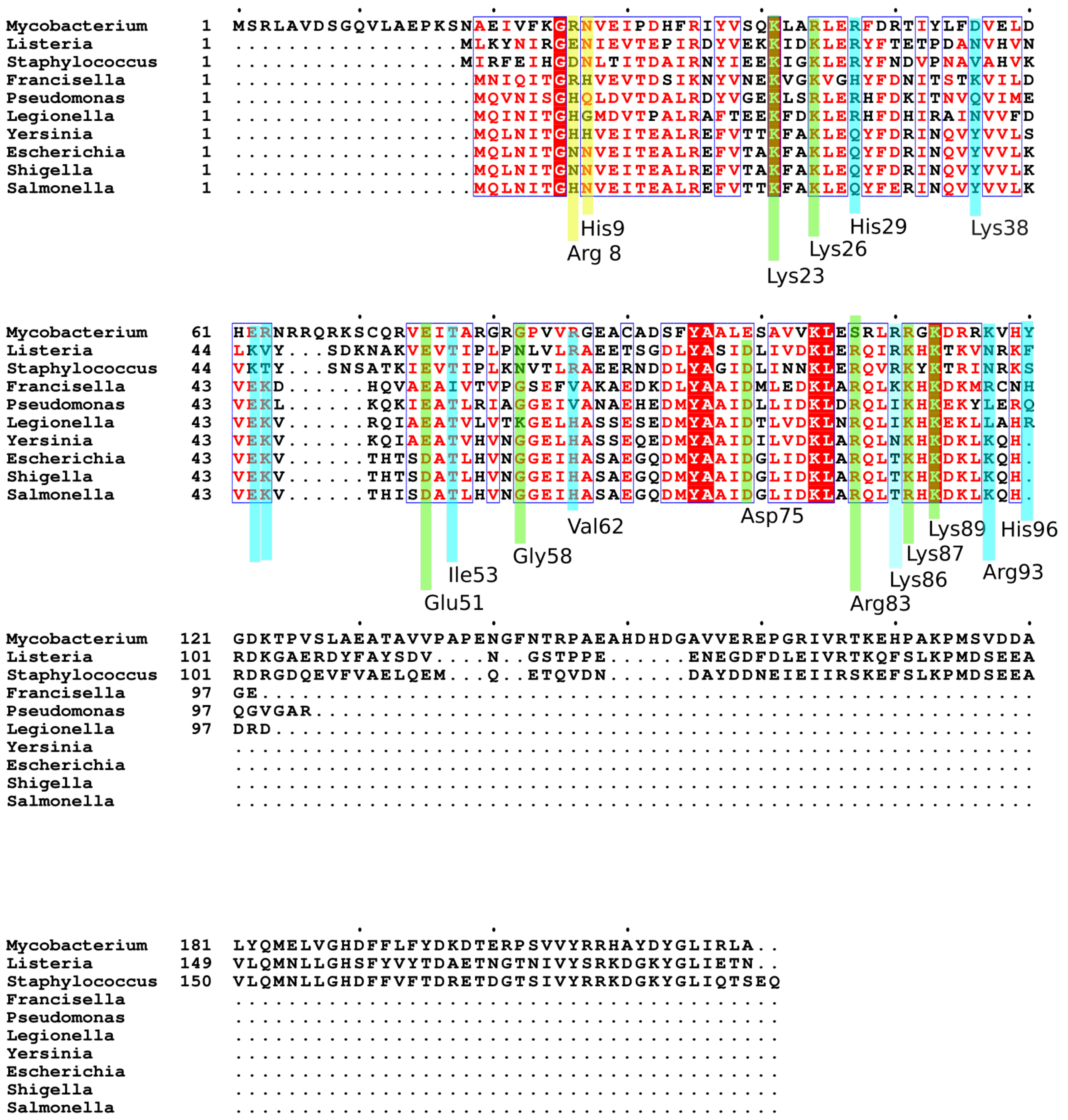

SI Figure 7. RaiA alignments

Sequence alignment of *F. tularensis* RaiA with homologous proteins from selected species. The residues responsible for interaction with 16S rRNA of 30S subunit are highlighted (green for high degree of homology, cyan and yellow for lower degrees of homology).

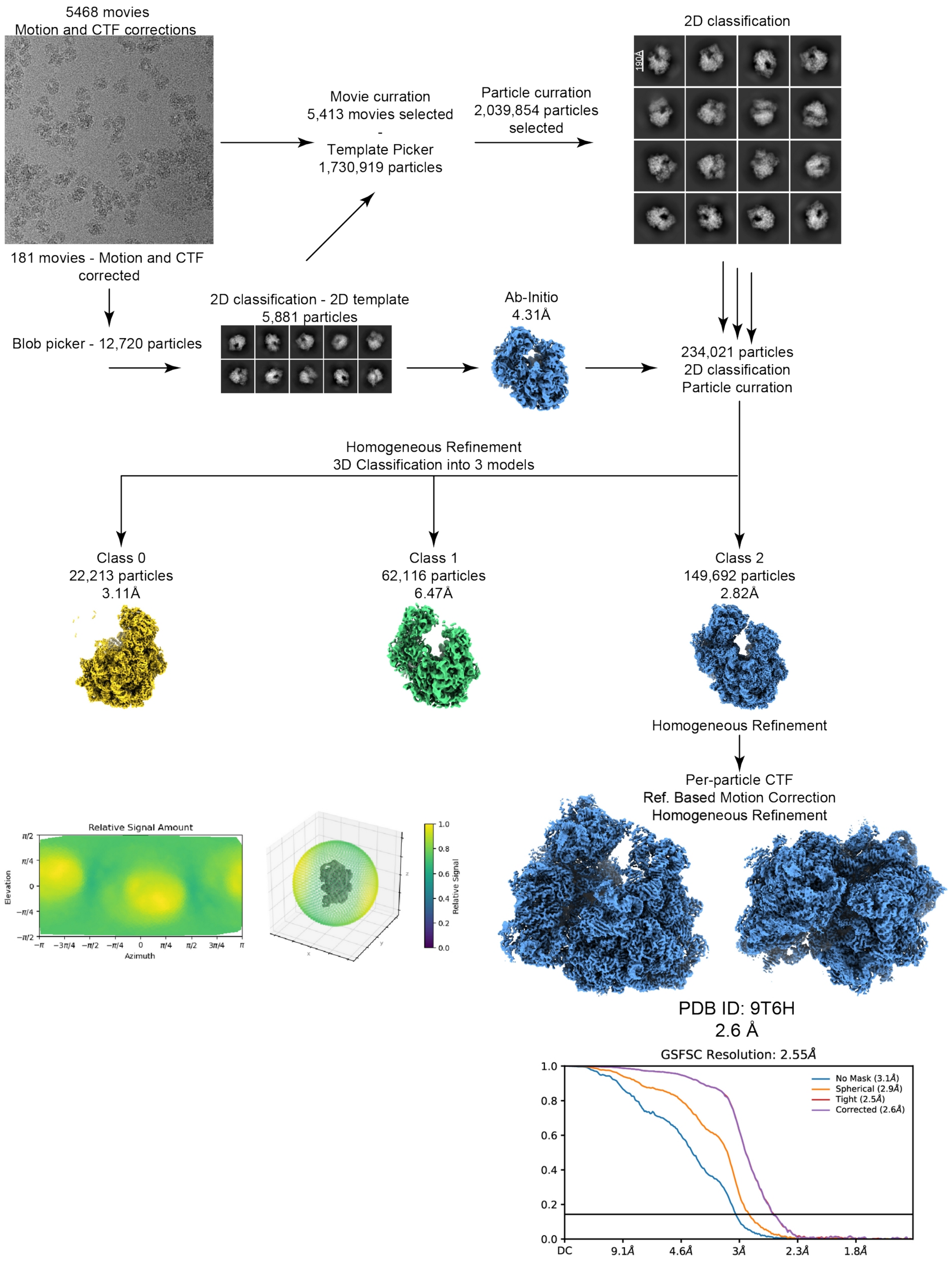

SI Figure 8. Workflow *F. tularensis* 70S with Antibiotics Cm&GEN
Cryo-EM processing of *F. tularensis* 70S with antibiotics chloramphenicol and gentamicin including the final 3D reconstruction with GSFSC final resolution.

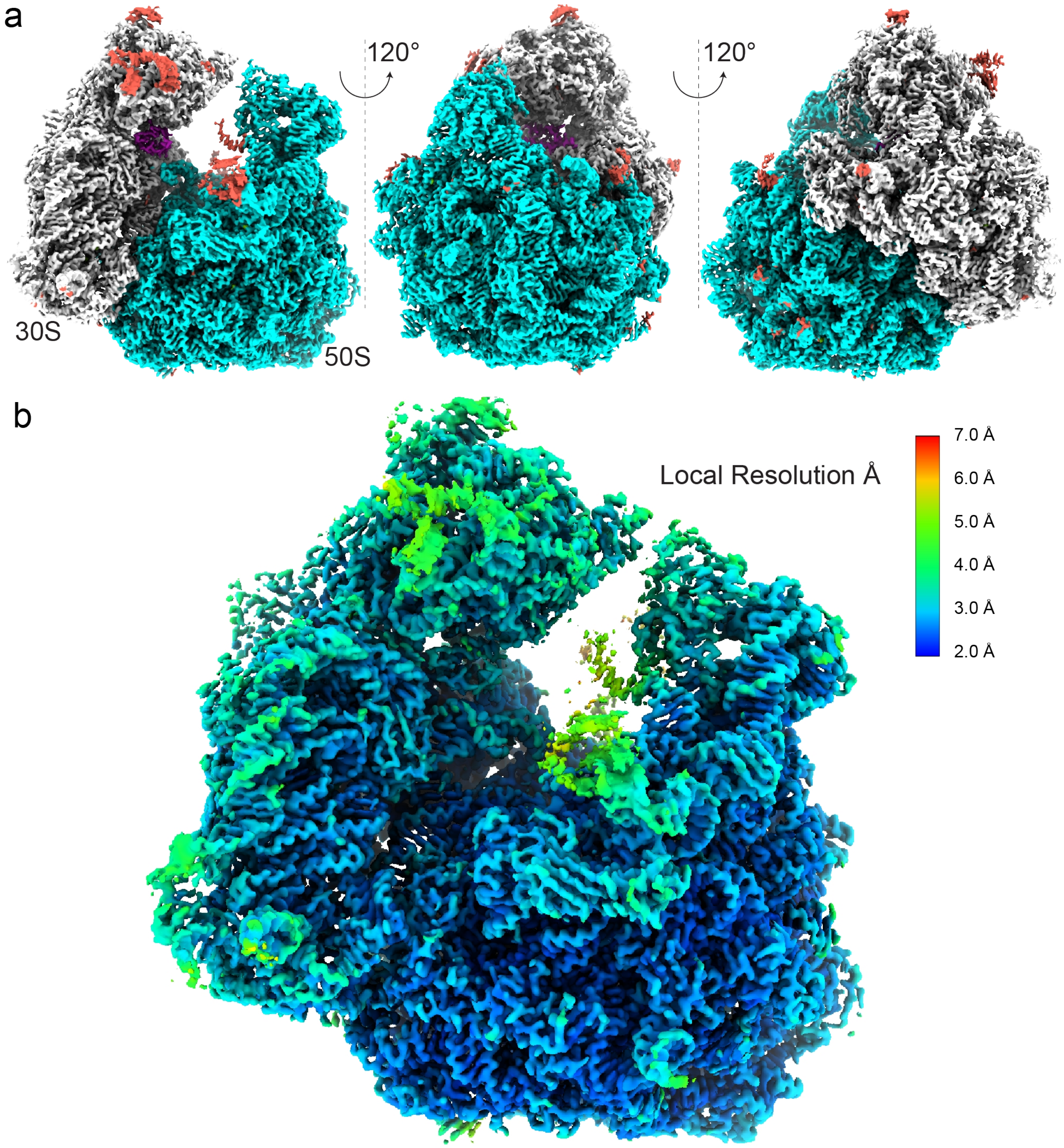

SI Figure 9. CryoEM maps of 70S ribosome with Antibiotics Chloramphenicol and Gentamicin (Cm&GEN)

a Cryo-EM maps coloured by chain, 30S white, 50S cyan, RaiA violet. Maps with poor density that did not allow model building are shown in red. **b** Cryo-EM map coloured by local resolution estimation.

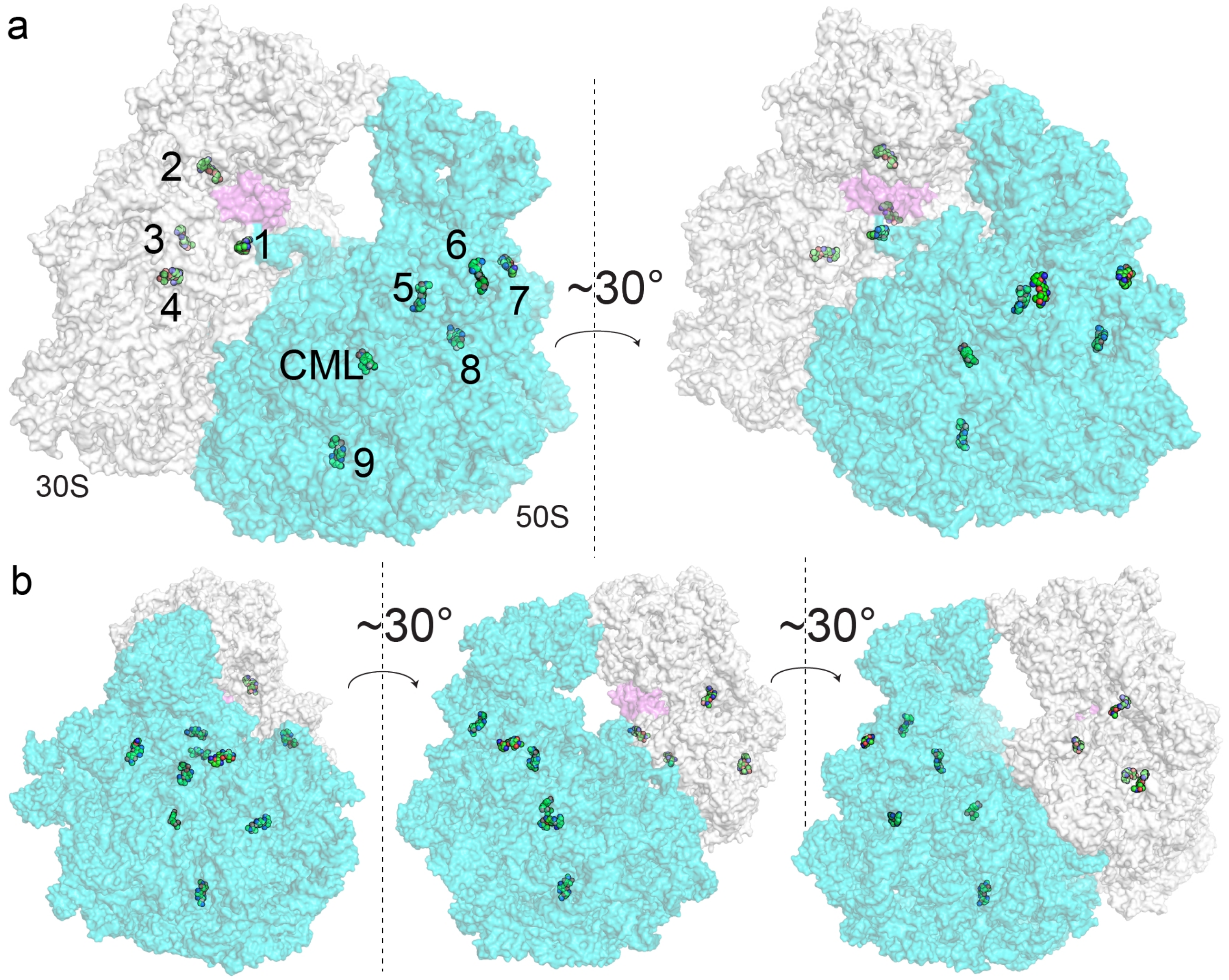

SI Figure 10. CryoEM maps of 70S ribosome with the location of all Antibiotics
a-b 70S ribosom with antibiotics shown as spheres, one chloramphenicol (CML) and nine GEN antibiotics shown, 70S is shown as surface representation 30S white, 50S cyan, RaiA violet.

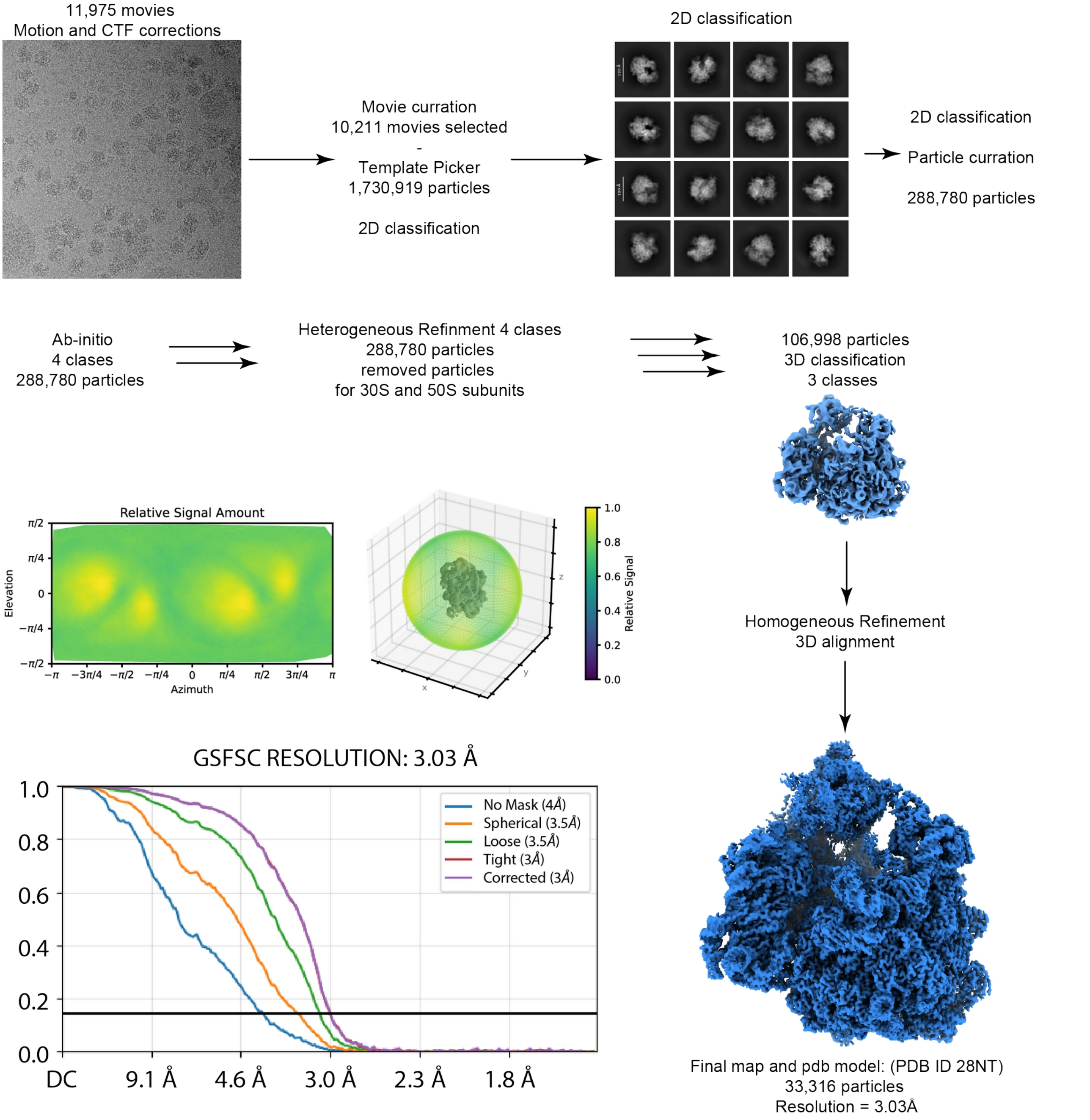

SI Figure 11. Cryo-EM workflow of *F. tularensis* RT ribosomes without RaiA

Cryo-EM processing of *F. tularensis* 70S prepared at room-temerature including the final 3D reconstruction.

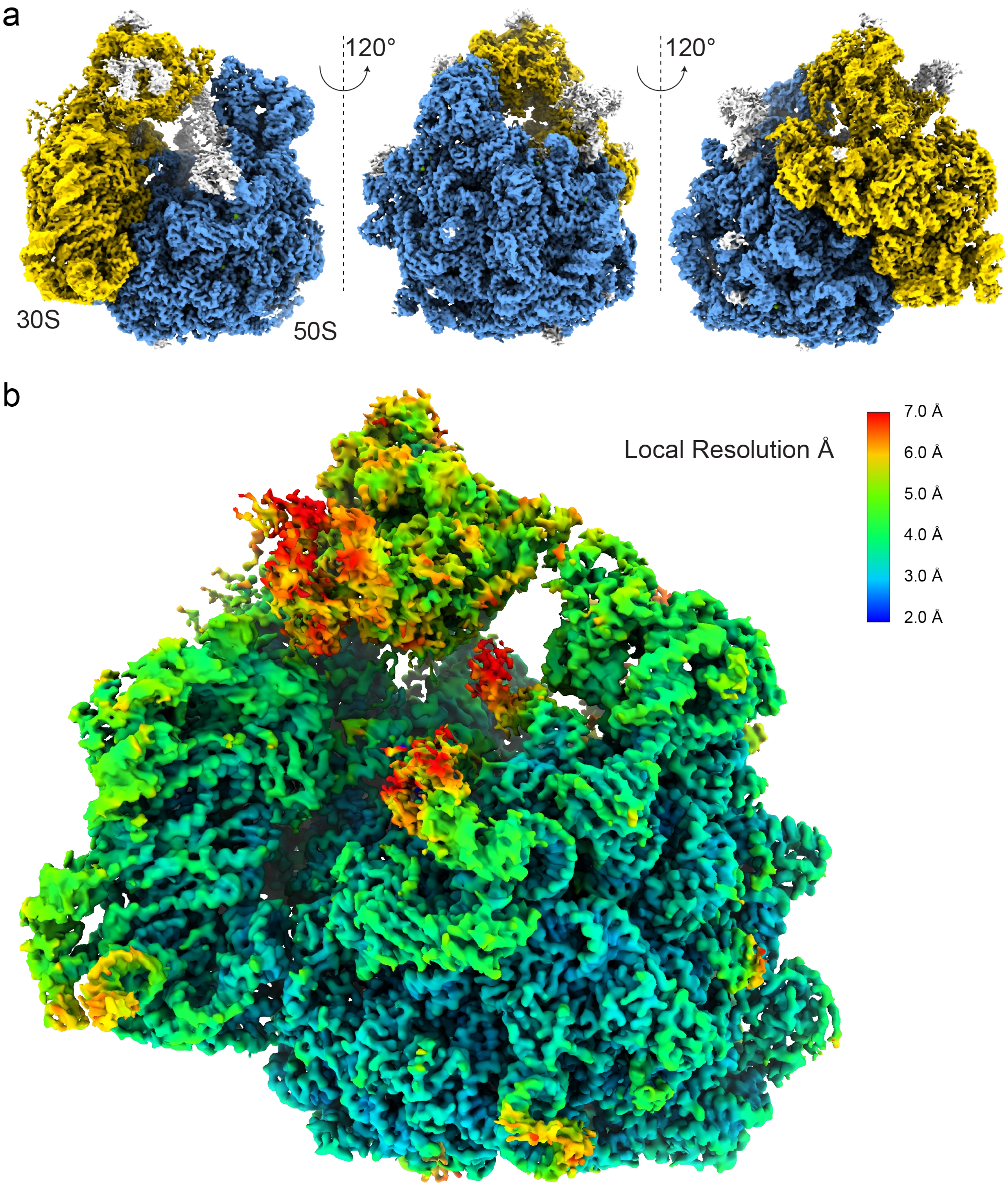

SI Figure 12. Cryo-EM maps of ft70S ribosome (28NT) and local resolution estimation
a Cryo-EM maps coloured by chain, 30S yellow, 50S blue, RaiA . Maps with poor density that did not allow model building are shown in white. **b** Cryo-EM map coloured by local resolution estimation.

Supplementary Table 1: Cryo-EM data collection, refinement and validation statistics.

	F. tularensis 70S (EMDB-54782) (PDB- **9SDA**)	*F. tularensis* 70S CM&GEN (EMDB-55615) (PDB- **9T6H**)
**Data collection and processing**		
Microscope	Titan Krios	Titan Krios
Detector	Falcon 4i	Falcon 4i
Magnification (nominal)	165.000x	165.000x
Voltage (kV)	300	300
Spherical aberration	2.7 mm	2.7 mm
Total electron dose (e–/Å^2)	40	60
Defocus range (μm)	-3.0 to -0.8	-3.0 to -0.8
Pixel size (Å)	0.76	0.76
Stage tilt	0°	0°
Number of Micrographs	28,005	5468
Final particle images (no.)	476,383	149,692
Map resolution (Å) [FSC threshold]	2.50 [$FSC_{0.143}$]	2.55 [$FSC_{0.143}$]
**Refinement**		
Initial model used (PDB code)	9SDA	9T6H
Symmetry during reconstruction	C1	C1
RMSD		
Bond lengths (Å)	0.007	0.007
Bond angles (°)	0.817	0.818
Validation		
MolProbity score	1.10	1.12
Clashscore, all-atom	1.80	1.93
Rotamer outliers	0.55%	0.55%

Ramachandran plot		
Favored	97.07%	97.07%
Allowed	2.93%	2..93%
Outliers	0.00%	0.00%

**Model vs. Data**		
Ligands (no.)	0	10 (1xCm, 9xGEN)
$CC_{unsharpened}$ (mask/box/ligand)	0.93 / 0.86 / --	0.86 / 0.82/ 0.74

Supplementary Table 2: Cryo-EM data collection, refinement and validation statistics of room-temperature F.tularensis 70S.

	F. tularensis 70S (EMDB-56659) (PDB- 28NT)
**Data collection and processing**	
Microscope	Titan Krios
Detector	Falcon 4i
Magnification (nominal)	165.000x
Voltage (kV)	300
Spherical aberration	2.7 mm
Total electron dose (e–/Å^2)	40
Defocus range (μm)	-3.0 to -0.8
Pixel size (Å)	0.76
Stage tilt	0°
Number of Micrographs	11,211
Final particle images (no.)	33,316
Map resolution (Å) [FSC threshold]	3.03 [$FSC_{0.143}$]

**Refinement**	
Initial model used (PDB code)	9SDA
Symmetry during reconstruction	C1
RMSD	
Bond lengths (Å)	0.004
Bond angles (°)	0.554
Validation	
MolProbity score	1.33
Clashscore, all-atom	3.49
Rotamer outliers	1.09%
Ramachandran plot	
Favored	97.09%
Allowed	2.91%

Outliers	0.00%

**Model vs. Data**	
Ligands (no.)	Zn: 1; Mg: 153
$CC_{unsharpened}$ (mask/box/ligand)	0.91 / 0.84 / 0.89

Supplementary methods

Preparation of room temperature Ribosomes: Ribosomes were prepared as described previously, with minor modifications. *Francisella tularensis* subsp. *holarctica* FSC200 was cultivated in BHI medium supplemented with 0.1% cysteine and harvested at $OD_{600} \approx 0.6$. In contrast to the previous protocol, all cooling steps were omitted prior to cell lysis, and the culture was processed as rapidly as possible without gradual temperature reduction. Cells were collected by centrifugation and washed with PBS at room temperature, and all centrifugation steps up to lysate preparation were performed at room temperature. Subsequent ribosome purification steps were carried out as described in the Materials and Methods section.

Cryo-EM preparation of RT (room temperature) ribosomes: The dataset for RT *F. tularensis* was collected and processed following the same procedure as the previous two datasets and under identical conditions. In more detail, Quantifoil R2/1 300-mesh copper grids were loaded with 2.5 μL of prepared RT ribosomal sample, vitrified in liquid ethane, clipped, and the dataset was collected as described above on Titan Krios. Processing of the antibiotic-bound 70S dataset followed an analogous workflow to that used for the other two ribosome datasets. The final map was obtained from 33,316 particles with an estimated resolution of 3.0 Å (GSFSC-0.143 criterion). The final maps and the atomic model were deposited in the Protein Data Bank and Electron Microscopy Data Bank under PDB ID 28NT (pdb_000028nt) and EMDB entry EMD-56659, respectively. For data collection and final model statistics see Supplementary Table 2, processing details SI Figure 11, final maps including the local resolution map see the SI Figure 12.
